# Caesarean section delivery and its associated factors in Ghana: A multilevel analysis

**DOI:** 10.1371/journal.pone.0318223

**Published:** 2025-02-12

**Authors:** Werkneh Melkie Tilahun, Mulat Belay Simegn, Alegntaw Abate, Eyasu Bamlaku Golla, Mohammed Ahmed Ali, Hawi Kumbi, Smegnew Gichew Wondie, Habtamu Geremew

**Affiliations:** 1 Department of Public Health, College of Medicine and Health Sciences, Debre Markos University, Debre Markos, Ethiopia; 2 College of Health science, Oda Bultum University, Chiro, Ethiopia; 3 Department of Midwifery, College of Health Science, Oda Bultum University, Chiro, Ethiopia; 4 Department of Laboratory, Adama Hospital Medical College, Adama, Ethiopia; 5 Department of Human Nutrition, College of Medicine and Health Science, Mizan Tepi university, Mizan Aman, Ethiopia; Medical Research Council, SOUTH AFRICA

## Abstract

**Background:**

Even if the ideal rate for caesarean section (CS) is considered 10–15%, worldwide CS rates have been steadily increasing, without significant additional benefit to women’s health. Its overuse was associated with maternal and fetal complications. Therefore, this study aimed to determine the rate of CS delivery and associated factors among women delivered in health facilities in Ghana.

**Methods:**

A cross-sectional study was conducted using the 2022 DHS datasets from Ghana. A total of 5231 weighted samples were included. STATA.16 was used for analysis. A multilevel logistic regression was applied. An adjusted odds ratio with a 95% CI and a p-value <  0.05 was used to declare significantly associated factors.

**Result:**

The prevalence of CS in Ghana was 20.29% [95% CI = 19.23–21.41%]. Age 25-34 [AOR = 1.32, CI: 1.04, 1.68] and 35 + [AOR = 1.57, CI: 1.16, 2.11], primary [AOR = 1.58, CI: 1.21, 2.07], secondary [AOR = 1.36, CI: 1.07, 1.73], and higher [AOR = 2.24, CI: 1.58, 3.17] education, richer [AOR = 1.45, CI: 1.06, 1.99] and richest [AOR = 2.35, CI: 1.63, 3.39] wealth index, employment [AOR = 0.77, CI: 0.64, 0.92], primiparous [AOR = 1.33, CI: 1.07, 1.67], giving first birth after the age of 35 [AOR = 4.58, CI: 1.88, 11.19], multiple pregnancy [AOR = 5.53, CI: 4.02, 7.62], large birth size [AOR = 1.25, CI: 1.06, 1.48], 4–6 kg birth weight [AOR = 2.13, CI: 1.55, 2.92], as well as residing in Volta [AOR = 1.98, 95% CI: 1.22, 3.22] and Bono East [AOR = 2.02, 95% CI: 1.28, 3.19] regions were significant factors associated with CS delivery in Ghana.

**Conclusion:**

The rate of CS was higher than previous studies in Ghana and the WHO recommended level, making it a public health concern. Age, education, wealth, employment, parity, age at first birth, pregnancy type, birth size, birth weight, and region were significantly associated with CS. Therefore, strategies aimed at examining guidelines for proper indications and uses of CS may lead to an improvement CS rate.

## Introduction

The rate of cesarean sections (CS) has increased globally, from 7% in 1990 to 21.1% nowadays [1]. If current trends continue, by 2030, the highest rates are expected to be found in Eastern Asia (63%), Latin America and the Caribbean (54%), Western Asia (50%), Northern Africa (48%), Southern Europe (47%), and Australia and New Zealand (45%) [1]. Even if CS is vital to saving lives in situations where vaginal deliveries would pose risks, not all the caesarean sections carried out at the moment are needed for medical reasons [1,2].

Mothers’ rising requests for CS were the main reason for the rapid increase [1–3]. In one study, the preference for a cesarean section was reported by 26% of participants [4]. The policies and funding of the health sector, cultural norms, beliefs, and behaviors, the number of premature deliveries, and the standard of care are among the drivers [1]. Nevertheless, the doctor’s convenience or preference has been the primary motivator behind the mother’s desire for CS [2]. These factors will likely result in an increase in the global caesarean section rate over the next ten years, with nearly one-third (29%) of all deliveries expected to occur via CS by 2030 [1].

Although CS is a necessary and life-saving procedure, it can place women and babies at higher risk of short- and long-term health complications if performed without a medical necessity [1]. The rate of CS has been rising globally without significant additional improvement to either the health of the mothers or their babies [5]. Rather, its overuse was associated with maternal and fetal complications, including health and behavioral problems [1,6–8]. Neonates delivered by CS were more likely to experience complications such as higher rates of neonatal intensive care unit (NICU) admission [6,9,10], low appearance, pulse, grimace, activity, and respiration (APGAR) scores [11], increased mortality [11,12], childhood obesity [13], breathing problems [11], increased mortality [11,12], and childhood obesity [13]. Additionally, complications related with caesarean sections include postpartum hemorrhage [6], preeclampsia [14], headaches, hip pain following delivery, difficulty with daily activities, breathing issues, recurrent infections, reduced food demand, fewer hours of sleep, and difficulties with breastfeeding [7].

Even if the international health-care community has considered the optimal rate of CS to be between 10% and 15%, studies in different countries have reported steadily increasing rates [5]. For instance, a study conducted in Jordan indicated that about 50.5% of the deliveries over 18 months were by CS, of which about 60% were planned procedures [6,9]

The causes of high caesarean section usage vary within and across countries [1]. It is also indicated that there are strong geographical inequalities in caesarean births in Ghana [15,16], which range between 4 and 26.9% [8,11,15–19]. This rate of CS was followed by a high rate of fetal mortality (76.4 per 1000 births) following the procedure [11].

Previous studies conducted in different areas revealed that primary and above education [17–20], being unmarried [17], wealth quintiles from poorer to richest [12,17–19,21], attending antenatal care (ANC) services [17,20,21], history of termination of pregnancy [4,17], being multigravida [18,19,21], reporting prolonged or obstructed labour [18], history of CS delivery [22–24], poor satisfaction with the previous intrapartum care [4], poor knowledge about CS delivery [4], urban residence [4,20,23], pregnancy-related problem including ante partum hemorrhage and mal-presentation [4,23], multiple pregnancy [20,21], and birth weight greater than 4000 gm [24] were factors significantly associated with increased CS rates. While other studies revealed gestational ages from 37 to 40 weeks [17], female baby sex [21], singleton pregnancy [24], being older than 24 years of age [19,20,22], birth weight less than 2500 gm [24], and parity of 2–4 children [20] were factors significantly associated with decreased CS rates. Another study also revealed that CS was related to factors such as older age, mother employment, low educational level, diabetes mellitus, preeclampsia, anemia, previous history of stillbirth, previous history of CS, breech fetal presentation, and twin pregnancy [25].

Even though research has been done in Ghana [13,16], studies based on national data are crucial to obtaining a nationwide overview of the issue and up-to-date information about the recent exponential growth in the CS rate. Therefore, the current study aimed to determine the magnitude of CS delivery and its associated factors among women who gave birth in health facilities in Ghana using recent GDHS data.

## Method and materials

### Data sources, setting, population, sampling and study design

This is a cross-sectional study and secondary data analysis conducted based on the 2022 Ghana Demographic and Health Survey (GDHS), which is the seventh survey conducted in Ghana. Data were collected from a nationally representative sample from all 16 regions in Ghana from October 17, 2022, to January 14, 2023. The survey interviewed 17,933 households and 15,014 women aged 15 to 49. The report provides information on fertility, maternal and child health, nutrition, HIV prevention methods, women’s empowerment, and other health issues. A sample of 18,450 households was selected from 618 clusters, which resulted in 15,014 interviewed women aged 15–49. The sampling procedure used was stratified, two-stage cluster sampling. A sampling frame prepared by the Ghana statistical service (GSS) based on the 2021 population and housing census was used. In the first stage, 618 target clusters were selected from the sampling frame using a probability-proportional-to-size strategy for urban and rural areas in each region. Then the number of targeted clusters was selected with equal probability through systematic random sampling. In the second stage, a household listing and map updating operation was carried out in all of the selected clusters to develop a list of households for each cluster. A fixed number of 30 households in each cluster were randomly selected from the list for interviews. More information about the survey can be found in the 2022 GDHS report [26]. We accessed the data from (http://www.dhsprogram.com) based on an online request.

### Participants

Our study was based on datasets from pregnancy and postnatal care recode (NR files). A total of 5231 weighted reproductive-age women who had experienced birth in the last three years were included. However, reproductive-aged women who didn’t have a birth in the past 5 years were excluded from the analysis.

## Variables of the study

### Outcome variable

The outcome variable was the CS, which was dichotomous and coded by the value “1” (one) if the respondents underwent CS and “0” (zero) otherwise.

### Independent variables

Both individual and community-level explanatory variables were included in this study. Maternal age, education, wealth, women media exposure, sex of household head, history of termination of pregnancy, health insurance, marital status, employment, sex of the child, acceptability of husband abuse, perceived difficulty of health care access, parity, age at first birth, modern contraceptive use, pregnancy type, pregnancy wanted when became pregnant, ANC visit, birth size, and birth weight were among the individual-level factors. Community-level characteristics, including residence and region, were evaluated.

### Measurement and operational definition

#### Household media exposure.

created by combining whether women read a newspaper or magazine, listen to the radio, or watch television and coded as “yes” (if a woman has been exposed to at least one of these media) or “no” (if she has not).

### Acceptability of spousal abuse

Attitudes towards spousal abuse were represented by five items. Women were asked whether they agreed with their husband’s beating in relation to wrongdoing such as going out without telling him, neglecting children, arguing with him, refusing to have sex with him, and burning food. The response options were yes, no, or I don’t know. “I don’t know why” responses were coded as missing and variables were reverse coded, so a woman scored “1” if she responded no, which shows greater autonomy and a more gender-equitable belief or attitude [27].

### Data management and analysis

The MEASURE DHS website provided the data. The statistical analysis was conducted using Stata version 16. Results were reported using text, figures, and tables, and descriptive and inferential statistics were used. Logistic regression analysis was anticipated as the result variable includes two binary possible responses (yes or no). This is not consistent with the homoscedasticity assumption since observations from the same clusters are not always independent. Since a multilevel regression model is expected to explain clustering effects, a multilevel logistic regression model was used to discover relevant components. The intraclass correlation coefficient (ICC) and median odds ratio (MOR) were used to measure the unexplained heterogeneity of the outcome. The variability in the odds of a caesarean section explained by successive models was estimated using the proportional change in variance (PCV) and calculated as follows:


$$\text{PCV}=\frac{\text{VA}-\text{VB}}{\text{VA}}*100$$

Where VA and VB are the neighborhood variance in the empty model and the variance in the successive models [28]. Sampling weight (v005/1000000) was an adjustment factor applied to each case in the tabulations to account for variations in the cases’ probabilities of selection and interview resulting from either sample design or chance.

### Model building process

An empty model served as the starting point for the process of developing more complicated models, step by step. Our study generated four models: model I, which is the null model; model II, which incorporates only individual-level factors; model III, which incorporates just community-level factors; and model IV, which incorporates individual and community-level factors. The deviance and information criteria (AIC) were used to evaluate the model’s comparison. To find the associated factors, two-level logistic regression models were fitted. Ultimately, significant factors were identified using adjusted odds ratios (OR) in the multivariable analysis of the chosen model, along with a 95% confidence interval (CI) and a p-value ≤ 0.05.

### Missing values

Any missing data in the dataset was handled appropriately as per the DHS rules. Thus, complete observation provided the basis for the final model.

### Ethical considerations

Our study was a secondary data analysis based on publicly available DHS datasets; participant consent and ethical approval were not necessary. However, we asked the MEASURE DHS program for the data, and we were given permission to download and utilize it.

## Results

A total of 5231 weighted reproductive-age women who had experienced birth in the last three years were included. Nearly one-fourth (24.47%) were within the age group of 25 to 29. More than half (52.19% and 53.62%) were rural residents and attained secondary level education, respectively. About 23.78% were from the poorest household wealth index. The majority (82.99%) reported that they had household media exposure. More than two-thirds (69.57%) reported males as household heads. Nearly three-fourths (73.67%) had not previously experienced termination of pregnancy. Almost all (95.04%) had health insurance. The majority (83.34%) of the participants were currently married. More than three-fourths (77.3%) were employed. More than three-fourths (78.15% and 80.3%) didn’t accept husband abuse and had at least four ANC visits, respectively. More than half (51.33% and 55.52%) had delivered male babies and revealed that they experienced difficulty accessing health care services, respectively. About 44.8% and 66.77% of women delivered a large and normal-weight baby, respectively (Table 1).

**Table 1:** Socio-demographic and obstetrics related characteristics of the study participants.

Variables	Category	Weighted Frequency	Percentage
Maternal age	15–19	287	5.49
	20–24	1072	20.51
	25–29	1280	24.47
	30–34	1241	23.71
	35–39	911	17.41
	40–44	348	6.65
	45–49	92	1.76
Education	No education	1147	21.93
	Primary	806	15.41
	Secondary	2805	53.62
	Higher	473	9.04
Wealth index	Poorest	1244	23.78
	Poorer	1098	20.98
	Middle	1041	19.89
	Richer	979	18.72
	Richest	869	16.63
Media exposure	No	890	17.01
	Yes	4341	82.99
Sex of household head	Male	3640	69.57
	Female	1591	30.43
History of termination of pregnancy	No	3854	73.67
	Yes	1377	26.33
Health insurance	No	259	4.96
	Yes	4972	95.04
Marital status	Never in union	594	11.36
	Currently in union	4360	83.34
	Formerly in union	277	5.31
Current working status	No	1187	22.70
	Yes	4044	77.30
Sex of the child	Male	2685	51.33
	Female	2546	48.67
Acceptability of husband abuse	No	4088	78.15
	Yes	1143	21.85
Perceived difficulty of health care access	No	2327	44.48
	Yes	2904	55.52
Parity	Primi-gravida	1301	24.88
	Multigravida	3930	75.12
Age at first birth	≤ 15	257	4.92
	16–18	1301	24.87
	19–24	2644	50.55
	25–34	981	18.74
	≥ 35	48	0.92
Modern contraceptive use	No	3672	70.19
	Yes	1559	29.81
Pregnancy type	Single	4967	94.94
	Multiple	264	5.06
Pregnancy wanted when became pregnant	Then	3213	61.43
	Later	1529	29.23
	No more	489	9.35
ANC visit	No	88	1.68
	1–3	463	8.85
	4 +	4202	80.33
	Don’t know/missing	478	9.14
Birth size	Large	2344	44.80
	Average	2218	42.40
	Small	650	12.44
	Don’t know	19	0.36
Birth weight	<2500 g	460	8.80
	2500–4000 g	3493	66.77
	4000–6000 g	240	4.59
	Not weighted/don’t know	1038	19.84
Residence	Urban	2501	47.81
	Rural	2730	52.19
Region	Western	321	6.14
	Central	537	10.27
	Greater Accra	639	12.22
	Volta	194	3.72
	Eastern	372	7.10
	Ashanti	925	17.69
	Western North	142	2.72
	Ahafo	116	2.22
	Bono	176	3.36
	Bono East	283	5.40
	Oti	181	3.46
	Northern	580	11.08
	Savannah	165	3.16
	North East	174	3.33
	Upper East	271	5.18
	Upper West	155	2.95

### Rate of caesarean section

**Fig 1:**
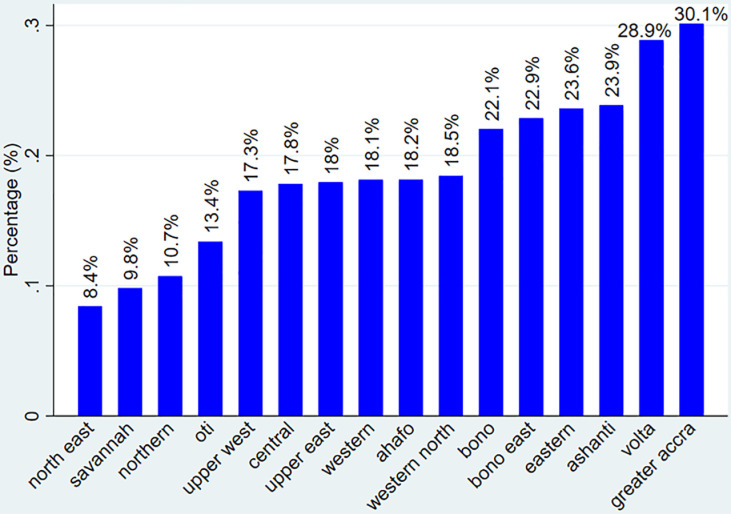
The rate of CS among women across regions of Ghana.

The rate of CS in Ghana was estimated at 20.29% with a 95% CI of [19.23–21.41%]. This indicates that one of the five women in Ghana delivered using a caesarean section. This rate was significantly higher among urban residents [26.49%, CI = 24.80%, 28.26%] compared to rural residents [14.61%, 95% CI = 13.34–15.99%], with a difference of 10.19% (p = 0.000). Regarding regional difference the highest (30.1%) and lowest (8.4%) of CS rates was observed in greater Accra and north east regions (Fig 1).

### Random effect analysis

The empty model showed that 14.45% [95% CI = 11.0, 18.75] of the variability in the odds of CS delivery in Ghana was explained by cluster level. A likelihood-ratio (LR) test comparing the model with classical logistic regression was also significant. The between-cluster level variability declined over successive models, from 14.45% to 4% in the individual-level only model, 6% in the community-level factors only model, and 3% in the combined model or final model (model IV). A likelihood-ratio (LR) test comparing the two-level model with the classical logistic regression was also significant (chibar^2^ (01) = 96.43, P = 0.0000). Thus, a multilevel approach is clearly favored over the classical approach and also over carrying out either of the potential two-level analyses. Moreover, the MOR was estimated at 2.04 in the empty model. This means if we pick two clusters randomly from two clusters with high and low rate of CS delivery and then move a woman from low to high rate areas, the probability of CS delivery increases by 2.04 fold. This estimate decreased to 1.34 in the final (Model IV) model. The PCV indicated that the addition of predictors to the empty model explained the variations in CS delivery. The variance estimates between clusters decreased from 0.56 in the empty model to 0.1 in the final model. The proportion of variance explained by the final model rose to 82.14%. Candidate multilevel models such as the null model (model I), model II (with individual level factors), model III (with community level factors), and model IV (with both individual and community level factors) were compared. Finally, model IV was selected since it showed a smaller deviance and AIC (Table 2).

**Table 2:** Comparison of model fitness measures among candidate multilevel models.

Model- Parameter	Model - I	Model – II	Model - III	Model - IV
AIC	5243.45	4740.84	5119.79	4736.14
Deviance (−2 LL)	5239.44	4662.84	5083.80	4626.14
MOR [95% CI]	2.04 [1.81, 2.26]	1.45 [1.23, 1.67]	1.54 [1.35, 1.74]	1.34 [1.11, 1.58]
ICC [95% CI]	14.45 [11.0, 18.75]	0.04 [0.02, 0.09]	0.06 [0.03, 0.10]	0.03 [0.01, 0.09]
PCV	Ref	73.21%	62.5%	82.14%
Variance	0.56	0.15	0.21	0.10

^a^AIC- Akaike Information Criteria; ICC- Intraclass Correlation Coefficient; LL- Log Likelihood; MOR- Median Odds Ratio; PCV- Proportional Change in Variance.

### Fixed effect analysis

Our study revealed that the odds of CS among women in the age ranges of 25–34 and 35 and above was 32% [AOR = 1.32, 95% CI: 1.04, 1.68] and 57% [AOR = 1.57, 95% CI: 1.16, 2.11] higher compared to those within the age range of 15 to 24. The odds of caesarean section among women with primary, secondary, and higher education was 1.58 [AOR = 1.58, 95% CI: 1.21, 2.07], 1.36 [AOR = 1.36, 95% CI: 1.07, 1.73], and 2.24 [AOR = 2.24, 95% CI: 1.58, 3.17] times higher compared to non-educated women, respectively. The odds of CS among women with the richer and richest household wealth index was 1.45 [AOR = 1.45, 95% CI: 1.06, 1.99] and 2.35 [AOR = 2.35, 95% CI: 1.63, 3.39] times higher compared to women with the poorest household wealth index, respectively. The odds of CS was 23% [AOR = 0.77, 95% CI: 0.64, 0.92] lower among working women compared to non-working women. Primiparous women had 33% increased odds of CS [AOR = 1.33, 95% CI: 1.07, 1.67] compared to multiparous women (Table 3).

**Table 3:** Results of bi-variable and multivariable multilevel logistic regression analysis of CS delivery among women in Ghana.

Variables	Category	COR [95% CI]	AOR [95% CI]
Maternal age	15–24	1.00	1.00
	25–34	1.52 [1.26, 1.84]**	1.32 [1.04, 1.68] *
	35 and above	1.63 [1.32, 2.02]**	1.57 [1.16, 2.11]**
Education	No education	1.00	1.00
	Primary	1.76 [1.37, 2.27]**	1.58 [1.21, 2.07]**
	Secondary	2.10 [1.71, 2.57]**	1.36 [1.07, 1.73] *
	Higher	6.52 [4.95, 8.59]**	2.24 [1.58, 3.17]**
Wealth index	Poorest	1.00	1.00
	Poorer	1.38 [1.09, 1.74] *	0.98 [0.76, 1.25]
	Middle	1.88 [1.48, 2.40]**	1.17 [0.87, 1.57]
	Richer	3.00 [2.34, 3.81]**	1.45 [1.06, 1.99] *
	Richest	5.99 [4.67, 7.70]**	2.35 [1.63, 3.39]**
Media exposure	No	1.00	1.00
	Yes	1.95 [1.57, 2.42]**	1.18 [0.93, 1.49]
Sex of household head	Male	1.00	1.00
	Female	1.23 [1.05, 1.45] *	1.04 [0.87, 1.25]
History of termination of pregnancy	No	1.00	1.00
	Yes	1.36 [1.15, 1.61]**	1.10 [0.93, 1.32]
Health insurance	No	1.00	1.00
	Yes	1.11 [0.78, 1.60]	0.71 [0.49, 1.04]
Marital status	Never in union	1.00	1.00
	Currently in union	0.94 [0.74, 1.20]	0.88 [0.66, 1.17]
	Formerly in union	1.03 [0.68, 1.56]	1.07 [0.69, 1.65]
Current working status	No	1.00	1.00
	Yes	0.94 [0.79, 1.12]	0.77 [0.64, 0.92]**
Sex of the child	Male	1.00	1.00
	Female	1.00 [0.86, 1.15]	1.00 [0.86, 1.17]
Acceptability of husband abuse	No	1.00	1.00
	Yes	0.60 [0.50, 0.72]**	0.89 [0.73, 1.09]
Perceived difficulty to healthcare access	No	1.00	1.00
	Yes	0.67 [0.58, 0.78]**	0.99 [0.84, 1.16]
Parity	Primi-gravida	1.25 [1.06, 1.47] *	1.33 [1.07, 1.67] *
	Multigravida	1.00	1.00
Age at first birth	≤ 15	1.00	1.00
	16–18	0.92 [0.62, 1.37]	0.91 [0.60, 1.38]
	19–24	1.14 [0.78, 1.67]	1.05 [0.70, 1.57]
	25–34	2.48 [1.67, 3.69]**	1.33 [0.86, 2.06]
	≥ 35	10.56 [4.61, 24.16]**	4.58 [1.88, 11.19]**
Modern contraceptive use	No	1.00	1.00
	Yes	1.38 [1.18, 1.61]**	1.36 [1.15, 1.60]**
Pregnancy type	Single	1.00	1.00
	Multiple	4.35 [3.29, 5.75]**	5.53 [4.02, 7.62]**
Pregnancy wanted when became pregnant	Then	1.00	1.00
	Later	0.97 [0.82, 1.15]	1.02 [0.85, 1.23]
	No more	1.05 [0.79, 1.40]	1.11 [0.82, 1.50]
ANC visit	No	1.00	1.00
	1–3	1.39 [0.56, 3.45]	1.07 [0.43, 2.68]
	4 +	3.12 [1.32, 7.37] *	1.61 [0.68, 3.85]
	Don’t know/missing	2.96 [1.22, 7.17] *	1.09 [0.44, 2.71]
Birth size	Large	1.19 [1.01, 1.40] *	1.25 [1.06, 1.48]**
	Average	1.00	1.00
	Small	1.26 [1.00, 1.59] *	1.23 [0.96, 1.59]
	Don’t know	0.37 [0.08, 1.61]	0.77 [0.17, 3.41]
Birth weight	< 2500 g	1.72 [1.36, 2.16]**	1.35 [1.02, 1.77] *
	2500–4000 g	1.00	1.00
	4000–6000 g	2.11 [1.55, 2.86]**	2.13 [1.55, 2.92]**
	Unweighted/don’t know	0.34 [0.26, 0.43]**	0.51 [0.40, 0.67]**
Residence	Urban	2.08 [1.75, 2.49]**	1.10 [0.91, 1.34]
	Rural	1.00	1.00
Region	Western	1.00	1.00
	Central	0.99 [0.59, 1.65]	1.06 [0.65, 1.71]
	Greater Accra	2.02 [1.24, 3.315]**	1.34 [0.84, 2.14]
	Volta	1.90 [1.13, 3.18] *	1.98 [1.22, 3.22]**
	Eastern	1.43 [0.86, 2.40]	1.41 [0.87, 2.29]
	Ashanti	1.49 [0.92, 2.43]	1.31 [0.83, 2.06]
	Western North	1.01 [0.59, 1.71]	1.19 [0.72, 1.97]
	Ahafo	1.01 [0.60, 1.70]	1.31 [0.80, 2.14]
	Bono	1.33 [0.79, 2.24]	1.48 [0.91, 2.43]
	Bono East	1.42 [0.87, 2.30]	2.02 [1.28, 3.19]**
	Oti	0.72 [0.43, 1.21]	1.15 [0.70, 1.89]
	Northern	0.55 [0.33, 0.90] *	0.91 [0.56, 1.50]
	Savannah	0.48 [0.29, 0.81]**	0.88 [0.54, 1.46]
	North East	0.40 [0.24, 0.67]**	0.81 [0.49, 1.36]
	Upper East	1.00 [0.61, 1.65]	1.32 [0.82, 2.12]
	Upper West	0.93 [0.56, 1.55]	1.27 [0.78, 2.06]

^a^Level of significance at * p < 0.05; **p < 0.01.

Women who gave their first birth after the age of 35 had 4.58 times higher odds of caesarean section [AOR = 4.58, 95% CI: 1.88, 11.19] compared to women whose age was less than 15 years during their first birth. Previous history of modern contraceptive utilization had increased the odds of caesarean section by 36% [AOR = 1.36, 95% CI: 1.15, 1.60] compared to their counterparts. Women with multiple pregnancies had 5.53 times higher odds of CS [AOR = 5.53, 95% CI: 4.02, 7.62] compared to those who gave birth to singletons. Women who gave birth to babies with large birth size and less than 2500 g birth weight had a 25% [AOR = 1.25, 95% CI: 1.06, 1.48] and a 35% [AOR = 1.35, 95% CI: 1.02, 1.77] increased odds of caesarean section compared to average birth size and normal birth weight (2500–4000 g), respectively. The odds of CS was 2.13 [AOR = 2.13, 95% CI: 1.55, 2.92] times higher among women who gave birth to a child with a birth weight of a birth weight of 4000 to 6000 g. The odds of CS delivery among women who were residing in the Volta and Bono East regions were 1.98 [AOR = 1.98, 95% CI: 1.22, 3.22] and 2.02 [AOR = 2.02, 95% CI: 1.28, 3.19] times higher compared to women residing in the western region of Ghana, respectively.

## Discussion

The overall rate of CS delivery in Ghana was 20.29% [95% C = 19.23–21.41%]. This indicates the rate of CS in Ghana has surpassed the ideal acceptable 10–15% of the CS rate stated by the WHO [1]. Thus, CS becomes a public health concern in Ghana based on the WHO classification of the public health relevance of CS [5]. Our study also revealed that the odds of CS was significantly associated with women’s age, education, wealth, employment, parity, age at first birth, modern contraceptive utilization, pregnancy type, birth size, birth weight, and region.

One in five women in Ghana have been delivered by caesarean section (20.29%, 95% CI [19.23–21.41%]). This finding is in line with the recent global estimate (21%) [1,2,29]. According to the WHO, the global trend is set to continue to increase by up to 29% by 2030 [1]. The rate of CS delivery in this study was lower than that for Latin America and the Caribbean (42.8%) [30], as well as Vietnam [31]. However, the current finding was higher than that estimate for whole Africa (9.2%) and sub-Saharan Africa (5%) [30]. Moreover, the current estimate was higher than the previous studies conducted in Rwanda (15.6%) [21] and Ethiopia (6%) [20]. In addition, the current estimate was higher than the previous studies conducted in Ghana: 6.59% [32], 11.4% [19], and 14.6% [17]. This might be due to time differences in the studies since one of the regions with the expected increase in CS rate was sub-Saharan Africa [30].

The odds of CS among women in the age ranges of 25–34 and 35 and above were higher compared to those within the age range of 15 to 24. This finding is consistent with a study conducted in Ethiopia [20], Mozambique [33], Vietnam [31], Ghana [17,32], and Egypt [34]. This might be due to the effect of advanced maternal age on pregnancy outcomes. It is indicated that mothers aged 25–29 years had lower risks of CS, while advanced maternal age was associated with higher risks of comorbidities like gestational diabetes mellitus, CS, hypertension, low birth weight, hypertensive disorders of pregnancy, preeclampsia, placenta accreta, placenta previa, preterm birth, large for gestational age, macrosomia, and fetal congenital anomaly [35–37].

Moreover, aging and its associated physiological and anatomical changes expose older mothers to high-risk pregnancy complications at delivery. It is also known that pregnancy-related problems can highly increase the likelihood of women’s preference for cesarean delivery [4,25] in protecting the fetus [17,19]. CS is considered an appropriate intervention for antepartum hemorrhage, prolonged or obstructed labor, pre-eclampsia, and intrapartum fetal distress [38]. In relation to this, our study also identified that women who gave birth to babies with less than 2500 g birth weight and women who gave their first birth after the age of 35 had increased odds of CS compared to younger and normal birth weight pregnancies. This is due to the fact that low birth weights are significantly associated with complications during pregnancy and delivery, including CS [39]. Due to prenatal compromise, such as failure to advance in labor, fetal distress, non-reassuring heart rate, and a low Apgar score, women who gave birth to LBW are at a higher risk of cesarean delivery [40,41].

Formally educated women had higher odds of CS compared to formally non-educated women. Previous studies conducted in Ghana [17,19,32], Ethiopia [20], Mozambique [33], and Bangladesh [42] reported similar findings. This might be related to the effect of education on the enhancement of maternal health care services, including CS delivery. Education is often associated with better utilization of prenatal health care services [43–45], where potential complications can be identified early, leading to a higher likelihood of CS recommendations by healthcare providers. Informed decision-making about their health and childbirth is high among educated women [46,47]. Thus, educated women may feel more confident in discussing their birth plan with healthcare providers and opting for CS if they believe it aligns with their preferences or medical needs. Moreover, since CS is associated with higher costs related to medical bills, hospital stays, and follow-up care, educated women can afford the cost compared to their counterparts. In relation to this, our study also found higher odds of a CS among women with the richer and richest household wealth index compared to women with the poorest household wealth index. Other previous studies conducted in Mozambique [33] and Ghana [17,19,32] also clearly indicated that women with a better wealth index were more likely to undergo CS deliveries.

The odds of CS was lower among working women compared to non-working women. Given that working women, particularly those with part-time jobs, were less likely to undergo a cesarean delivery in order to return to work quickly, this may have something to do with the time required to return to formal work [48]. Additionally, working women might share duties and receive greater social support and health-related information from their coworkers regarding the benefits and drawbacks of CS delivery, which could aid in their decision-making. This finding is consistent with a previous report from Bangladesh [42]. Another study conducted in Iraq [25] also found a significant association between CS and women’s employment status. However, a study conducted in Ghana [19] and Ethiopia [4] indicated that there was no association between preferences for CS employment status.

Primiparous women had increased odds of a caesarean section compared to multiparous women. This finding is also supported by a previous study conducted in Ghana [8,19,32], Mozambique [33], and Egypt [34]. This might be due to fear or unwillingness to bear labor pain [42].since primiparous women were nulliparous (had not given birth before) at the actual time they had CS. Furthermore, the rate of obstetric complications was higher in primiparous mothers as compared to multiparous mothers [49]. However, another study conducted in Ghana [17] reported that women with two or more births were more likely to deliver by CS compared to those with only one child.

Women with multiple pregnancies had higher odds of a caesarean section compared to women who gave birth to singletons. This finding was in line with previous reports from sub-Saharan Africa [12], Ethiopia [20], Rwanda [21], and Egypt [34]. This might be due to the effect of multiple pregnancies on pregnancy and labor-related complications like preterm labor, premature rupture of membranes, malposition and malpresentation, gestational diabetes, gestational hypertension, preeclampsia, and intrahepatic cholestasis, all of which may increase the likelihood of giving birth via CS [4,20].

In our study, the odds of a caesarean section was higher among women who gave birth to a child with less than 2500 and 4000 to 6000 g birth weight. This finding is in line with a study conducted in Vietnam [31] (where birth weights of < 2.5 kg and over 3.5 kg) and Egypt [34] (where birth weights of < 2.5 kg and over ≥ 4 kg) had an increased likelihood of CS. Another study conducted in Ethiopia indicated that a birth weight > 4 kg was significantly associated with an increased risk of CS [24]. In contrast to our finding, a similar study conducted in Ethiopia indicated that birth weight < 2.5 kg was related to a reduced risk of CS [24]. From our findings, not only higher than average birth weights are associated with CS, but also low birth weights. This might be due to the effect of low birth weight on complications during pregnancy and delivery [39].

Women with a history of modern contraceptive use had increased odds of CS compared to their counterparts. This might be the effect of modern contraceptive utilization on maternal age at delivery. Modern contraceptive utilization significantly delays childbearing and birth space, which can lead to a higher maternal age at the time of delivery [50,51]. Our study also indicated that women who gave birth after the age of 35 had increased odds of CS. This is due to the effect of advanced age on the increased risk of complications during pregnancy and delivery, potentially leading to higher CS rates [52].

The odds of CS delivery among women who were residing in the Volta and Bono East regions were higher compared to women residing in the western region of Ghana. This finding is consistent with previous reports from Ghana [15,16]. This might be due to socioeconomic and demographic differences across regions of Ghana [19,32,53].

### Strength and limitation of the study

Our study’s primary strength is the use of nationally representative, population-based samples gathered using a validated instrument that encompasses both rural and urban areas. However, there are certain constraints to our study. A number of behavioral and lifestyle-related factors may be related with the issue. However, we are unable to investigate their effects because of absence of these variables in the dataset.

## Conclusions

The rate of CS delivery in the study population was higher than previous studies conducted in Ghana and WHO-recommended ranges. Factors such as age, level of education, wealth index, employment status, region, parity, age at first birth, modern contraceptive use, pregnancy type, birth size, and birth weight were significantly associated with CS delivery in Ghana. The Ghana Health Service has adopted the national CS guidelines developed by the Society of Obstetricians and Gynecologists of Ghana (SOGOG), whose members include many fellows of the Ghana College of Physicians and Surgeons’ Obstetrics and Gynecology department. At the moment, the guidelines are being used as a blueprint for obstetrics and gynecology resident training. They will also be very beneficial for teaching medical students and non-specialist physicians how to perform CS. Therefore, the Ghanaian Ministry of Health should develop strategies or policies that address modifiable sociodemographic and obstetric factors, examine and address guidelines for proper indications, and ensure that these guidelines have been met.
